# The Role of Support and Communication on Postpartum Pain: A Qualitative Analysis of Patient Experiences

**DOI:** 10.1111/birt.70023

**Published:** 2025-09-26

**Authors:** Julia D. DiTosto, Tazim Merchant, Karolina Leziak, Lynn M. Yee, Nevert Badreldin

**Affiliations:** ^1^ Division of Maternal‐Fetal Medicine, Department of Obstetrics and Gynecology Northwestern University Feinberg School of Medicine Chicago Illinois USA; ^2^ Department of Biostatistics, Epidemiology and Informatics Perelman School of Medicine, University of Pennsylvania Philadelphia Pennsylvania USA; ^3^ University of Michigan School of Medicine Ann Arbor Michigan USA

## Abstract

**Background:**

Postpartum pain, a common symptom after a cesarean birth, is influenced by psychosocial factors. This exploratory qualitative study examined patient perspectives on social support and healthcare communication behaviors in the postpartum setting in relation to the pain experience.

**Methods:**

In‐depth, semi‐structured, qualitative interviews about postpartum pain experiences were conducted 2–3 days and 2–6 weeks postpartum with individuals who underwent a cesarean birth (2020–2021). Data were analyzed using the constant comparative method.

**Results:**

Among 49 postpartum individuals, themes related to social support and healthcare communication were identified in relation to postpartum pain. Participants discussed the impact of non‐healthcare social support (e.g., partners, extended family, other children) on postpartum pain, highlighting emotional and practical assistance. Most commonly mentioned were the positive impacts of emotional and logistical support with household activities and childcare on postpartum pain recovery. The second theme covered individuals' views on how healthcare support and communication affected postpartum pain, with themes of both positive and negative experiences. Some participants discussed positive experiences of shared decision‐making and responsiveness of the healthcare team, whereas others recounted negative experiences of lack of counseling and poor outpatient communication.

**Discussion:**

Social support and healthcare communication are integral influences on pain recovery after a cesarean birth. These findings highlight the need for interventions to address psychosocial support and healthcare team communication in the immediate postpartum period.

## Graphical abstract

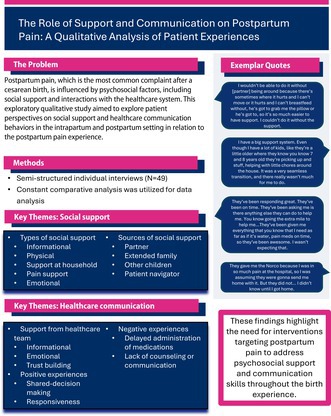

## 1 Introduction

Psychosocial support, including social support from relationships and healthcare team communication, improves health outcomes by helping patients cope with stressors [1, 2, 3, 4]. These forms of support enhance care access, emotional well‐being, informed decision‐making, and patient autonomy [4]. Such support is beneficial during the postpartum period [5, 6, 7]. For instance, postpartum individuals have voiced that receiving social support was essential to their physical and emotional recovery postpartum [5]. Positive interactions with healthcare teams, such as effective communication and counseling, have been shown to improve patient activation and symptom resolution [8, 9]. Conversely, poor healthcare communication experiences have been identified as a barrier to receiving adequate care during birth and the postpartum period [6].

Less is understood about how social support and healthcare communication impact postpartum pain. Inadequate pain control not only hinders recovery, but also increases risks of complications such as postpartum depression [10]. Given the simultaneous challenge of managing parental responsibilities with recovery, psychosocial support may be crucial for improved postpartum pain control, and a deeper understanding can guide tools and strategies to enhance postpartum recovery. Thus, we aimed to better characterize patient perspectives on social support and healthcare communication behaviors in relation to the postpartum pain experience.

## 2 Methods

This was a secondary analysis using data from a prospective, qualitative investigation evaluating postpartum individuals' experiences with pain and perspectives on opioid use in postpartum pain management. The methods for the primary analysis have been described previously [10]. Briefly, inclusion criteria included English‐ or Spanish‐speaking pregnant or postpartum individuals aged > 18 years old with publicly funded insurance who received their prenatal care at an urban academic medical center and had a cesarean birth after 20 weeks' gestation. Exclusion criteria included contraindications to opioid or non‐steroidal anti‐inflammatory drugs (NSAIDs), ≥ 3 opioid prescriptions in the year prior to birth, receipt of general anesthesia intrapartum, admission to the intensive care unit, or a known substance disorder [11]. Recruitment occurred from 11/2020 to 10/2021.

Participants underwent two interviews led by a trained research member fluent in the participant's preferred language (English or Spanish): (1) 60‐min face‐to‐face interview conducted between postpartum days 2 and 3 (“inpatient interview”); and (2) 20‐min phone interview conducted between 2 and 6 weeks after hospital discharge (“discharge interview”). Interviewers used semi‐structured interview guides to facilitate open‐ended, non‐judgmental conversation about the postpartum pain experience and opioid use. Interviews included questions related to preparation for postpartum pain, prenatal and postpartum communication with the healthcare team regarding the birth experience, and barriers and facilitators to recovery. Participants were contacted 2 weeks after hospital discharge to schedule the discharge interview. Loss‐to‐follow‐up was defined as 5 unsuccessful attempts of communication. Participants were compensated for each completed study activity. All interviews were audio‐recorded and professionally transcribed. Spanish interviews were translated to English, and all interviews were deidentified prior to analysis. Transcripts were uploaded into Dedoose, a secure web‐based tool for data management and qualitative coding [12].

The constant comparative method was used to analyze transcripts [13, 14, 15]. The study team created an initial codebook based on discussions regarding preliminary data, the interview guide, and background literature. The codebook was continuously revised based on the emerging findings and review of transcripts independently by three study team members (J.D.D., K.L., N.B.). Once a revised set of themes was developed, coding categories were developed and revised. Two study team members (J.D.D., K.L.) finalized the codebook and systematically applied to each interview transcript independently. Iterative discussions were used to organize, collapse, and refine the codebook as data analysis was performed. Results are structured by emergent themes, which are ideas that are not predetermined but are identified through the constant comparative method. Subthemes are displayed within the emergent themes and described using exemplary quotations.

This study was approved by the Institutional Review Board and registered on http://clinicaltrials.gov [16]. All participants provided written informed consent.

## 3 Results

Forty‐nine individuals completed an inpatient interview; 81.6% (*N* = 40) completed the discharge interview. The majority of participants identified as non‐Hispanic Black (*N* = 20, 40.8%) or Hispanic (*N* = 20, 40.8%) race and ethnicity, reported living with a partner (*N* = 16, 32.6%) or being married (*N* = 16, 32.6%), and had less than a college education (*N* = 37, 75.5%) (Table 1).

**TABLE 1:** Participant characteristics in the prospective qualitative study (*N* = 49).

Participant characteristics	*N* (%) or mean (SD)
Sociodemographic characteristics	
Age, years	33.3 (6.3)
Race and ethnicity	
Non‐Hispanic Black	20 (40.8)
Hispanic	20 (40.8)
Hispanic Black	3 (6.1)
Non‐Hispanic White	5 (10.2)
Asian	1 (2.0)
Relationship status	
Unpartnered	17 (34.7)
Living with partner	32 (65.3)
Education	
Less than college	22 (44.9)
Associate's degree or some college	15 (30.6)
College graduate	11 (22.4)
Primary language	
English	45 (91.8)
Spanish	4 (8.2)
Pregnancy characteristics	
Nulliparous	17 (34.7)
BMI upon admission, kg/m^2^	38.8 (9.2)
Gestational age at birth, weeks	38.3 (1.7)
Characteristics of cesarean birth^a^	
Primary cesarean	20 (40.8)
Scheduled cesarean	26 (53.1)
Labor prior to cesarean	23 (46.9)
Emergent cesarean	3 (6.1)

^a^ Characteristics of cesarean birth are not mutually exclusive.

Interview data were organized into two domains: themes related to non‐healthcare social support (Table 2) and healthcare support and communication regarding postpartum pain (Table 3).

**TABLE 2:** Themes related to non‐healthcare social support for postpartum pain.

Themes	Subthemes	Exemplary quotation
Types of social support	Informational support	My mom said just to be prepared for how much it's gonna hurt, like you're not going to be able to get up from the bed as much as you would want to sometimes. And she said both of her C sections were different. She said with my sister, she was like quick recovery, and then with my brother, it was more like, it was harder to get out of bed and take showers and stuff like that. My friends…said the same, one of them actually got an infection, so she was telling me how that was. And then the others were just telling me about how much pain they were in during the recovery. And …what they did to like take care of it basically. [inpatient quote]
	Physical support	I wouldn't be able to do it without [partner] being around because there's sometimes where it hurts and I can't move or it hurts and I can't breastfeed without, he's got to grab me the pillow or he's got to, so it's so much easier to have support. I couldn't do it without the support. [inpatient quote]
	Support with household activities and childcare	I have my significant other, my boyfriend and then his mom comes like once a week to help out, she comes over to cook for me or bring some food and she has been doing the grocery shopping since I'm not able to do it right now…He took over the responsibility of taking care of my daughter while I was in pain and then when I needed to go to emergency room he stayed home all night to take care of the baby by himself. [discharge quote]
	Support with pain management	My husband is literally giving it to me. He's the one that gives me the pills when I have to take them, so that I don't have to get up all the time in the middle of the night. [discharge quote]
	Emotional support	Just because in the sense that you're home. You know what I mean? You're more comfortable. I have more support there obviously too. Not more support, I take that back, because they're amazing here. I'll say I'll feel more open to ask for help from family. [inpatient quote]
Sources of social support	Partner/significant other support	I'm very blessed that I have my family's support and my husband's support. He's working from home. He was on paternity leave for two weeks after the baby so I wasn't having to do much and I still don't have to do much so that helped. [discharge quote]
	Extended family	… my daughter is doing e‐learning, so we made plans for her to stay with her grandparents. Her dad's parents for two weeks. And I will be at my mom house for two weeks. Because my husband's steady working, due to COVID, he's in and out of the house, and stuff like that. [inpatient quote]
	Other children	I have a big support system. Even though I have a lot of kids, like they're a little older where they know you know 7 and 8 years old, they're picking up and stuff, helping with little chores around the house. My daughter is out of college right now visiting but she too helps me with feedings here and there. [discharge quote]
	Patient navigator	Because [my navigator] supports me a lot. She helps me through a lot of stuff… Through everything… she asked me how I'm feeling and if my pain is managed. Because she'll ask me, I feel very comfortable with her because she asks me all the time do you need me to call anyone for you so she does all, I guess I want to say my dirty work (laughs). Because to me it's dirty work because I get nervous to call and she knows that so she takes that, she eases that away from me so all that burden on me I want to say she takes all that away. She's amazing actually. [inpatient quote]

**TABLE 3:** Themes related to healthcare support and communication regarding postpartum pain.

Themes	Subthemes	Exemplary quotation
Support from healthcare team	Emotional support	I'm glad that… they listened to me. And you know one of the nurses listened and she helped. And they also too, what I like is they know it's personal to me, so if they can, they make it personal. Like the nurse just, she had told me, she had shared with me some experience that she had in her own life with pregnancy. And how she felt about it, that she could relate. Then you know, some things that she had focused on to kind of not, you know, be so caught up in the idea of having the C‐section. [inpatient quote]
	Informational support	She [nurse] been helping me a lot, because I've been asking her stuff like some things that I don't know what to do, and maybe answering my questions and like I've asked like other people, they good like attitude, they been helping me… [inpatient quote]
	Trust building	I like the whole care team. To me it rehearses, go over everything, they do a self‐check like make sure this person is doing right and make them accountable to have an overall quality, so even though I went through all this extra experience, it was a good one in the end because it could have been worse. You do have nice people that genuinely care or are concerned about you and your baby's welfare. [inpatient quote]
Positive experiences with healthcare communication	Shared decision making	They have been really good. When I told them…that the epidural was helping me with just the incision but not the pain inside, they offered me options and they said look, it might help you, it might just make it a little bit worse, it's up to you, we can try it. Like they gave me options, it wasn't just like oh well you have to, like this is what you have. [inpatient quote]
	Responsiveness of healthcare team	They've been responding great. They've been on time. They've been asking me is there anything else they can do to help me. You know going the extra mile to help me…They've been given me everything that you know that I need as far as if it's water, pain meds on time, so they've been awesome. [inpatient quote]
	Satisfaction with outpatient interpersonal communication	Well, she emailed me a few days later to check on me and I just said I had pain and the symptoms I was going through and she told me to do the one‐time Tylenol, the other time ibuprofen and if it's still not getting better I should let her know so she can put in a prescription for the Norco for me. [discharge quote]
Negative experiences with healthcare communication	Delayed administration of medications	I'd just say ask me more often. I don't really know their protocol, but sometimes throughout the day the pain could be more extreme than most. Like I said I don't know how restricted I am with the prescriptions and how often they're allowed to give it to me, but other than that. I'd say they ask too little. I would want more. [inpatient quote]
	Lack of counseling or preparation for postpartum pain management	No, actually they didn't. About what to expect? Not really, no. They told me about what type of pain medicine or regimen they were gonna put me on to counteract the pain but they didn't get into specifics. So, once I did give birth, then I started noticing they would give me a certain medicine and then so many hours later they would give me another medicine. It wasn't explained in depth. Let's just say that much. But they did do a little bit of explaining. [inpatient quote]
	Lack of outpatient interpersonal communication	Well, I think on Thursday they gave me the Norco because I was in so much pain at the hospital, so I was assuming they were gonna send me home with it. But they did not… I didn't know until I got home. I emailed my doctor like the pain is too much and she said okay, she was gonna put the Norco in or the Oxy in but after I said I don't know the pharmacist texted me that my prescription was ready for pickup but I don't know if she put it in but I just waited out the pain. [discharge quote]

### 3.1 Themes Related to Non‐Healthcare Social Support

Participants discussed the influence of social support on postpartum pain; this domain addressed how non‐healthcare team members assisted participants emotionally or in a practical manner. Both the presence and absence of social support was addressed. Themes included specific types of social support and sources of social support (Table 2).

#### 3.1.1 Types of Social Support

The most prominent types of social support mentioned were informational support, physical support, support with household activities and childcare, support with pain management, and emotional support.

Informational support consisted of information received from family and friends regarding their experiences with pain after cesarean birth. When asked how the physical experience of pain compared to what she expected, a participant answered, “well, my sisters have already had c‐sections and…they had told me that the recovery takes longer than in a natural birth because, well, a c‐section is always 7 layers…that are cut. So, I knew that it was more painful than a normal birth.” Similarly, participants expressed an openness to receiving information from multiple sources to increase their understanding. Another participant recalled, “Family and friends, my doula, midwives… anybody that I could get information from while I was completing my birth plan throughout my pregnancy, that's who I used.”

Physical support included somatic assistance due to limited mobility from postpartum pain. Examples of physical support involved receiving physical help to move around and to feel comfortable. A similar type of social support was support with household activities and childcare, which included assistance with taking care of other children and completing chores (e.g., grocery shopping, cleaning). Related to childcare, multiple participants noted that their extended family was able to watch their other children in the immediate days upon returning home. One such participant said, “My mother‐in‐law was actually off of work for like two weeks so that helped out so much…So… I made arrangements for her [elder daughter] to stay with my mom.”

Support with pain management included reminding the individual to take pain medication and providing non‐pharmacological alternatives to pain management, such as heating pads or abdominal binders. A participant described, “My mom uses heating pads for all her various pain in her neck and back, so when I told her I was in pain… she gave me her heating pad…and it helped a lot.” Likewise, a participant mentioned the help she received: “I would tell my husband about it if I was in pain and he would help me get my medication or get my heating pads and stuff.”

Emotional support included consistent check‐ins and reassurance throughout the recovery process. One participant described the impact of emotional support she received from her family while inpatient:

I've had my daughter here and my husband here. There's a lot of women who don't have that though so I wonder what they do…you have your nurses that they do what they're supposed to do which is make sure that you take your meds correctly, that you're taken care of physically, but what about you emotionally taking care of your child…I really feel bad for someone who doesn't have that.

### 3.2 Sources of Social Support

Sources of social support included one's partner, extended family, other children, and patient navigators.

The most common source of social support was one's partner. One participant described the emotional support she received from her partner: “I have…a great partner who says you don't have to do that; you don't have to do this alone.” Conversely, another participant described the frustration with the lack of parental leave from her husband's job:

…it just sucks because my husband, it actually took him out of work but he's not getting paid for it and it just really sucks that they make it so hard on us…to not have a significant other…It's not fair at all.

Support from extended family such as one's parents or grandparents was common. One participant commented: “My mother was here so thankfully I was fortunate to have someone here with me. But not everyone is, so then I think about that too, I'm like oh wow what if she wasn't here…” Participants who had older children reported receiving social support from them. For example, one participant reported “It [pain] affected so much that I didn't even do much at all…I call my daughter for everything…She's 16.”

Finally, this study was a supplement to a larger trial in which participants were randomized to a patient navigator or usual care (see Methods). Six participants (12.6%) in this study had a patient navigator, who was mentioned as a source of social support. This subtheme was identified as the impact of support from the patient navigator. One such participant stated, “she's [patient navigator] amazing. She checks on me constantly.”

### 3.3 Themes Related to Healthcare Support and Communication

The second domain includes themes regarding participants' perspectives on the impact of healthcare support and communication on postpartum pain. The healthcare team was defined as bedside nurses, certified nurse midwives, attending and trainee physicians. Themes included support from healthcare team, positive experiences with healthcare interpersonal communication, and negative experiences with healthcare interpersonal communication.

#### 3.3.1 Support From Healthcare Team

Subthemes related to support from the healthcare team included emotional support, informational support, and efforts related to trust building.

Multiple participants recounted how their healthcare team provided emotional support while they were inpatient recovering from birth. Support from providers included feeling listened to and spending ample time addressing questions and concerns regarding pain. For instance, when asked about how her healthcare team has impacted her postpartum recovery process, one participant noted, “They're very special. They listen. That's what you look for… You look for someone who's compassionate, who's able to listen to your concern. And give you a good solution.”

Informational support differed from interpersonal communication and was specifically was defined as the healthcare team providing information, advice, guidance, or knowledge to help with aiding decision‐making and solving problems. One participant described how her healthcare team alleviated her anxiety:

…my anxiety is triggered from the not‐knowing…I could go in and just tell myself like, okay we know this is gonna happen and this is how it's gonna go and this is what you'll be on and this is how you'll feel… they were very informative about everything, so I felt really good about that.

Participants discussed the importance of having trust in their providers and building relationships. Some of this trust stemmed from institutional trust or previous birth experiences at this institution. One such participant said, “…I trust them, this institute more than others definitely. And I think their rapport with people play a big part in that.” Strategies participants affirmed were trust‐building included empathetic communication, frequent check‐ins, quick responsiveness to calls, and help with unexpected challenges in postpartum recovery. One participant noted:

Certain things that they do I don't think that it's part of their job description…they just go the extra mile. Like the other day…one of the nurses helped me in the bathroom…putting on my underwear and changing my pads. Probably her job, but it's just like, dang, that's, that's the extra mile…That's big (laughs), to help someone like that…

#### 3.3.2 Positive Experiences With Healthcare Communication

Subthemes related to positive experiences with healthcare communication included shared decision making, responsiveness of the healthcare team, and satisfaction with outpatient communication.

Participants expressed positive reactions to the incorporation of shared decision making in their communication with the healthcare team. When presented with the stepwise multimodal approach to postpartum pain management, one participant particularly appreciated her ability to choose when to take oxycodone for her pain:

I feel like it's been a good combination, because I can choose when to skip the Oxy. When I feel like the Tylenol and the Motrin are helping me on their own, I just don't take the Oxy. Once the pain gets to a certain level…I'll ask them for the Oxy.

Similarly, participants appreciated the responsiveness of the healthcare team when they had suboptimal pain. One participant detailed, “I just call her [nurse]…I'm not feeling well. She's like okay I'm on my way. I'll give you something right now.”

Participants were asked about outpatient communication with their healthcare team during the discharge interview. One participant recalled an instance when she called the nurses in the outpatient clinic with a question regarding her pain:

…I was a little skeptical about calling…and kinda not wanting to call. And, and then calling and you know, feeling you know good about it, it really makes the experience all the better.

#### 3.3.3 Negative Experiences With Healthcare Communication

Subthemes related to negative experiences with healthcare communication included delayed administration of medications, lack of counseling for postpartum pain management, and lack of outpatient communication.

Multiple accounts of delayed administration of medications leading to fluctuating pain were addressed by participants. One participant recounted a particularly difficult time inpatient:

…if they [nurses] get really busy…you don't really see them as often and they might forget your time schedule when you have to take pain meds. Because that happened to me yesterday, where one was an hour late and I was in pain, and it took her another 30 min to get to me, so I was just like okay, I get it, she's busy…but at the same time, if you can't help me can you get someone else to help me?

Another participant stated,

I called her and told her [nurse] I was ready for my Motrin and she was out helping another patient get into a car…So I was like, okay, yeah, you sound busy, but at the same time…maybe you shouldn't disclose this to a patient, but I think she just wanted to let me know that she was really busy. But if that was the case, I probably would have felt more comfortable if she got someone else to come in and give me my medicine because I was…in pain.

Multiple participants felt like they received a lack of counseling or preparation for postpartum pain management. When asked what her healthcare team counseled her regarding postpartum pain management recovery, one participant answered, “Honestly, they didn't explain it to me. The only thing they bring me are the pills and that's it.” Another participant hypothesized that her lack of counseling was due to the normalization of postpartum pain among the healthcare team:

… they're used to it, see it every day or I don't know. They see something normal. But for someone that's going…for someone who's first time mom…it's…tough… I don't know I guess they're like that because they see it every day. They experience it. But it's different when you're going through it.

Specific to counseling on side effects of opioids for postpartum pain management, one participant noted, “They didn't explain exactly what the oxycodone was for or what, if there were any side effects. They just gave me six different pills, they just said okay this is the name of this, this, and that.”

Regarding the lack of outpatient communication, some participants expressed confusion on who to contact and dissatisfaction with waiting times to hear back from healthcare providers. One participant expressed dissatisfaction with needing to leave a voicemail rather than speaking directly with a provider:

I don't like that fact that if you are calling you have to leave a voicemail…I think that they should have some, at least one doctor just for those calls…I had to leave a message and then I had to wait 30 or 40 min on her to call back.

This subtheme was commonly mentioned with regards to confusion with their discharge prescriptions, specifically directions on taking specific medications (e.g., number of pills, how often) and the option for refills. A participant who was discharged over a holiday weekend detailed her experience being unable to get more pain medication:

I guess just being sent home with like only the five [oxycodone] pills and then it was [holiday] weekend and I couldn't get a hold of anybody, so I went the whole weekend without anything until I was able to contact my OB, so I was just really surprised I guess.

## 4 Discussion

This study explored the influence of social support and healthcare team communication on the postpartum pain experience after a cesarean birth. In this cohort, social support and healthcare communication were key aspects of the postpartum pain experience that positively or negatively impacted postpartum recovery experiences. Building more robust systems to enhance support and the quality of communication may be beneficial to improve the postpartum pain experience.

In the general medical literature, the presence of social support is known to positively impact health and well‐being [1, 2, 3, 17]. Although this has not been explored in depth for postpartum pain, social support is an essential component for the physical and emotional well‐being of postpartum mothers [5, 17, 18]. Social support manifests in many ways, ranging from emotional support to tangible support with household activities. Our finding that support from one's partner is particularly impactful aligns with other studies [5]. However, over one‐third of participants were unpartnered, and these individuals identified the absence of social support as a challenge. Particularly in the US, where paid family and medical leave is uncommon for partners [19, 20], the absence of social support is more common for individuals who experience greater health disparities and are at greater risk for postpartum morbidity [21, 22, 23]. Therefore, it may be particularly important to provide additional forms of support for individuals who are alone during the postpartum period to decrease the risk of poor pain recovery [7].

Compared to the prenatal period, the postpartum period receives less attention and sparser communication with healthcare providers, which may present difficulties for individuals at high risk of postpartum pain. Our results support that positive experiences with healthcare communication and support improve postpartum pain experiences, particularly when those experiences included healthcare team responsiveness and consistent communication. Conversely, negative experiences were centered around inconsistent or insufficient communication, which may be reflective of patients interacting less with the healthcare system after discharge. This finding aligns with previous qualitative work that examined challenges of low‐income individuals with healthcare interactions during COVID‐19. In this study, participants reported that delays in care, specifically difficulties navigating postpartum care, led to negative experiences resulting in anxiety and frustration [6]. While in‐person visits may be difficult during the early postpartum period, as they were during the COVID‐19 pandemic, electronic communication, such as email, phone calls, or patient portal messaging, and telehealth may provide streamlined opportunities for patient‐provider communication that may improve the pain experience.

Successful interventions to reduce postpartum opioid prescribing exist, including checklists, discharge calculators, and patient education videos [24]. However, whether these interventions improve the postpartum pain experience on the patient level, or ameliorate the disparities that exist, has yet to be explored. Since social support and healthcare communication are relevant psychosocial influences on the postpartum pain experience, intervention development should include training and reinforcement for interpersonal skills, such as basic bedside communication skills and empathy training, for healthcare professionals. In the literature, group‐based interventions for chronic pain have demonstrated greater improvements in pain, depression, and physical functioning [25]. As prenatal and postpartum interventions such as group prenatal care or patient navigation become more common, the influence of support and communication embedded in these interventions should consider addressing pain‐related outcomes. Physical pain and recovery management are essential for the postpartum pain recovery process, but the psychosocial aspect of recovery may provide an opportunity to address the multifactorial nature of pain.

This analysis has many strengths, including its in‐depth, prospective investigation over a longitudinal period within the postpartum period. Incorporating two time points allowed evaluation of experiences over time, improved capture of the changing dynamics throughout the immediate postpartum period, and limited recall bias. Secondly, the majority of data on the postpartum pain experiences and various psychosocial influences on pain are quantitative; this analysis centers the often‐overlooked patient perspective. However, there are limitations to note; as with all qualitative analyses, findings are intended to be hypothesis‐generating and are not widely generalizable. Nevertheless, our cohort predominantly included individuals who self‐identified as Black or Hispanic, whom data have demonstrated experience the greatest burden of postpartum pain and inequitable receipt of opioid prescriptions to manage pain [26, 27]. Additionally, there may be potential for recall and social desirability bias. We attempted to reduce concern for recall bias for the 2–6 weeks postpartum interview by scheduling it as close to 2 weeks as possible, but individuals who completed the interview closer to 6 weeks may have a greater concern for recall bias. Regarding social desirability bias, the interviews were conducted by employees of the university associated with the academic medical center, but not involved in patient care; thus, there is potential that participants may have underreported socially undesirable attitudes and behaviors and overly reported socially desirable attributes [28].

Overall, this study explored patient perceptions of the influence of social support and healthcare communication on the postpartum pain experience after a cesarean birth. Insights uncovered in this analysis underscore the importance of considering psychosocial influences when developing interventions to improve the postpartum pain experience.

## Conflicts of Interest

The authors declare no conflicts of interest.

## Data Availability

The data that support the findings of this study are available on request from the corresponding author. The data are not publicly available due to privacy or ethical restrictions.
